# Functional Consequences of CFTR Interactions in Cystic Fibrosis

**DOI:** 10.3390/ijms25063384

**Published:** 2024-03-16

**Authors:** Yashaswini Ramananda, Anjaparavanda P. Naren, Kavisha Arora

**Affiliations:** 1 Department of Pediatrics, Division of Pulmonary Medicine, Cincinnati Children’s Hospital Medical Center, Cincinnati, OH 45229, USA; yashaswini.ramananda@cshs.org; 2 Department of Medicine, Division of Pulmonary and Critical Care Medicine, Cedars-Sinai Medical Center, Los Angeles, CA 90048, USA

**Keywords:** cystic fibrosis, Cystic Fibrosis Transmembrane Conductance Regulator, CFTR interactors, single-cell analyses of CFTR, CFTR modulators

## Abstract

Cystic fibrosis (CF) is a fatal autosomal recessive disorder caused by the loss of function mutations within a single gene for the Cystic Fibrosis Transmembrane Conductance Regulator (CFTR). CFTR is a chloride channel that regulates ion and fluid transport across various epithelia. The discovery of CFTR as the CF gene and its cloning in 1989, coupled with extensive research that went into the understanding of the underlying biological mechanisms of CF, have led to the development of revolutionary therapies in CF that we see today. The highly effective modulator therapies have increased the survival rates of CF patients and shifted the epidemiological landscape and disease prognosis. However, the differential effect of modulators among CF patients and the presence of non-responders and ineligible patients underscore the need to develop specialized and customized therapies for a significant number of patients. Recent advances in the understanding of the CFTR structure, its expression, and defined cellular compositions will aid in developing more precise therapies. As the lifespan of CF patients continues to increase, it is becoming critical to clinically address the extra-pulmonary manifestations of CF disease to improve the quality of life of the patients. In-depth analysis of the molecular signature of different CF organs at the transcriptional and post-transcriptional levels is rapidly advancing and will help address the etiological causes and variability of CF among patients and develop precision medicine in CF. In this review, we will provide an overview of CF disease, leading to the discovery and characterization of CFTR and the development of CFTR modulators. The later sections of the review will delve into the key findings derived from single-molecule and single-cell-level analyses of CFTR, followed by an exploration of disease-relevant protein complexes of CFTR that may ultimately define the etiological course of CF disease.

## 1. Introduction

### 1.1. From Discovery and Diagnostics to Therapy in Cystic Fibrosis

Cystic fibrosis (CF) disease was first described by Dorothy H. Andersen in the 1930s. Almost six decades later, the gene that encodes for the CF transmembrane conductance regulator (CFTR) protein and is responsible for CF disease was identified and cloned [1,2,3]. Significant improvements in clinical diagnoses of CF and the availability of revolutionary CF modulators/drug treatment in the past decade have dramatically improved the survival rate of people with CF [4]. The CF modulators directly address the fundamental defects caused by mutations in the CFTR protein. Modulator response has been shown to be influenced by the cellular mechanisms that affect biogenesis, trafficking, and cell surface regulation of CFTR. The cellular signaling responsible for CFTR is typically orchestrated by specific protein–protein interactions. As such, it is imperative to evaluate and further investigate these interactions to comprehend why certain patients become nonresponsive or have limited responses to modulators, as well as the differential effect of modulators among CF patients and the need to develop alternative therapies for some patients. Additionally, the question remains about the long-term effects of modulator therapy in the responsive population and how CFTR regulation by cellular components and the complex cell-to-cell and cell-to-microenvironment interactions become different in the context of modulators and redefine the disease course of CF. 

### 1.2. History of Cystic Fibrosis

Dr. Dorothy H. Andersen first identified cystic fibrosis (CF) in 1938 while studying the autopsies of malnourished infants. Andersen published her clinical and pathological findings in 49 children with pancreatic fibrosis [5]. The significant pathological changes observed in these patients were as follows: (i) Pancreatic acinar tissue layer substituted by cysts lined on the epithelium layer and fibrous tissue surrounding them; (ii) severe respiratory infections such as pulmonary abscesses, bronchitis, and bronchiectasis; (iii) patients who died before the age of 1 year showed vitamin A deficiency; and (iv) intestinal, cystic, or pancreatic duct atresia.

Based on distinct cystic and fibrotic appearances in the pancreatic tissue, she coined the term cystic fibrosis of the pancreas and identified it as a disease entity distinct from celiac disease [5]. Soon after, the commonness of severe lung dysfunction in CF patients was recognized. Several studies identified that the mucus glands of these CF patients were clogged with thick, sticky mucus, in addition to having a significant effect on their pancreas [6]. In the early to mid-1900s, due to limited treatment and a lack of confirmatory early diagnosis of CF, more than 50% of CF patients would die in their childhood [4].

In the late 1950s, a critical clinical finding of excess salt in CF patients’ sweat led to the development of a test to measure the chloride content of sweat that remains a gold standard test to diagnose CF today [7,8]. In the 1960s, early clinical diagnosis of CF through sweat tests was key to strategizing the best treatment plans for CF patients. The treatment plan involved three main approaches: (i) Interventions to maximize airway clearance, which included postural drainage and chest percussion; (ii) dietary therapy and pancreatic enzyme replacement; and (iii) antimicrobial treatment for pulmonary lesions [9]. 

It was noted that the epithelia are the most active sites for CF disease, and there is a conspicuous chloride transport defect in CF patients’ lung epithelia. In 1989, a breakthrough discovery of Cystic Fibrosis Transmembrane Conductance Regulator (CFTR) as a CF causative gene by Drs. Lap-Chee Tsui, John Riordan, Francis Collins, and their collaborators commenced tremendous efforts directed at understanding the CFTR protein and how it causes CF disease and the development of therapeutics [1,2,3]. Following the cloning of CFTR and the availability of CFTR cDNA, several seminal discoveries were made regarding this protein, as mentioned here: (i) The lack of a cAMP-regulated apical membrane Cl^−^ conductance in CF epithelia that could be restored upon expression of normal CFTR expression, suggesting CFTR dysfunction to be the primary CF defect and that it is regulated by cAMP [10]; (ii) the CFTR channel activity requires phosphorylation of a unique regulatory domain and ATP binding and hydrolysis by the two nucleotide-binding domains (NBDs); (iii) the most common CF mutation caused by deletion of three base pairs encoding of phenylalanine (F508del) CFTR causes a protein misfolding defect; and (iv) F508del mutant is temperature-sensitive and traffics to the surface and retains significant function when the incubation temperature is reduced [11].

As of today, CF is identified as an autosomal recessive disorder, affecting one in every 3500 newborns in the USA. CF predominantly affects Caucasian people due to the higher prevalence of the F508del CFTR allele among this population [12,13,14]. The research in CF to this point has been foundational for the current state of therapeutics in CF, which we will discuss in later sections. 

### 1.3. Multi-Organ Disease Manifestations in CF

CFTR is a chloride channel at the apical surface of epithelial cells lining the airway, pancreatic duct, sweat duct, intestine, biliary tree, and vas deferens. Defects in the CFTR protein perturb electrolyte homeostasis across the epithelial membrane, and as a result, the epithelium is the primary site of disease in CF. Lack of CFTR results in inadequate hydration and the buildup of thick, viscous mucus in the airways. The CF lung then becomes a thriving environment for bacterial growth and colonization, and recurrent infections occur, which cause inflammation. This vicious process of infection and inflammation causes progressive lung damage, and most people with CF die of end-stage lung disease [15]. 

**Figure 1:**
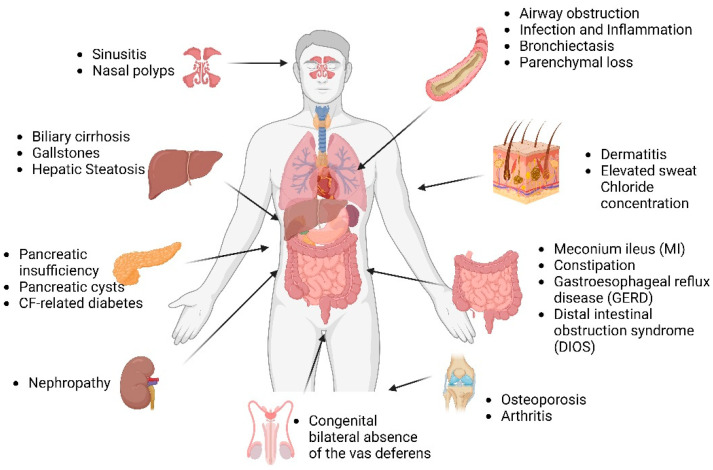
Multi-organ disease manifestations in cystic fibrosis.

Other than airway disease, intestinal obstruction, pancreatic insufficiency, congenital bilateral absence of the vas deferens, and biliary cirrhosis are major clinical features of CF [1,16] (Figure 1). There is a significant heterogeneity in CF as there are more than 2000 CFTR mutations, and there is further contribution by environmental factors and genetic modifiers to disease pathogenesis. With improved longevity, CF patients are likely to experience worse age-related morbidities, including diabetes, cognitive dysfunction, sinonasal disease, colorectal cancer, pancreatitis, chronic respiratory tract infections, and unexplained bronchiectasis.

#### 1.3.1. CF Lung 

Airway surface liquid (ASL) is a thin layer of fluid covering the epithelial surfaces that is crucial for mucociliary flow and the maintenance of an antimicrobial niche [17,18]. Hydration of ASL is regulated by the secretion and absorption of ions via channel transporters and protein pumps [19,20]. ASL is disrupted in CF due to a lack of CFTR-mediated chloride transport and fluid secretion [21,22]. Due to the lack of bicarbonate anion transport through CFTR, ASL pH is reduced in CF airways [23]. This is consistent across ASL samples taken from human primary airway epithelial cells, submucosal glands, human exhaled breath condensate, and tracheal ASL of newborn CF pigs [23,24,25,26]. However, the difference in pH levels between CF and non-CF individuals may vary depending on age and disease state. For instance, some studies have found no significant differences in ASL pH between CF and non-CF individuals aged three years or older [27], while others have reported lower nasal ASL pH in neonates with CF compared to non-CF neonates [28]. Interestingly, nasal pH levels in older CF children and adults were similar to those in individuals without CF [28]. Studies have demonstrated that the reduction in pH levels leads to a decline in the antimicrobial activity of ASL in CF pigs [26]. However, the precise mechanism(s) by which the pH level affects the ASL’s antimicrobial properties remains unknown at present. Depleted ASL in CF increases mucus burden due to reduced mucus clearance and enhanced mucus adhesion to the airway surface [29]. Thick mucus depositions on airway surfaces promote bacterial infection, colonization, and recurrent infections. Inflammation ensues from recurring infections leading to bronchiectasis, parenchymal loss, progressive airway destruction, and, in severe cases, complete lung function prolapse [21,30]. Nearly 30% of CF children develop bronchiectasis as early as three months of age, and about 60% of them by the age of 3 years [31]. Based on the data generated from large animal models of CF, it is now established that the CF become infected with bacteria at birth due to underlying host defense defects and that inflammation and airway remodeling appear later [32,33,34]. Several structural and developmental defects are also known to be present at birth in the CF airways [33]. 

#### 1.3.2. CF Pancreas

Although CF is commonly perceived as a lung disease, pancreatic dysfunction was the initial diagnostic criterion for CF [5]. Pancreatic insufficiency, pancreatic cysts, and CF-related diabetes (CFRD) are the major clinical complications of the pancreas in CF [35]. Pancreatic dysfunction in CF arises from multiple factors that include a lack of buffering from bicarbonate secretion from CFTR, insufficient fluid causing the accumulation of thick mucus in pancreatic ducts, and pro-inflammatory secretions [36,37]. Up to 90% of CF patients develop pancreatic insufficiency [38]. The formation of acinar plugs filled with zymogen granules and ductal obstruction cause fibro-inflammatory changes and parenchymal injury in the CF pancreas [39]. Abdominal bloating, greasy stools, flatulence, and inadequate weight gain are all clinical signs of pancreatic insufficiency [40]. Individuals suffering from cystic fibrosis have reduced exocrine enzyme secretion, which leads to maldigestion [41]. This causes a range of symptoms, such as steatorrhea, deficiencies in fat-soluble vitamins, and failure to thrive. Over time, CF patients develop diabetes mellitus as there is progressive fibrotic damage extending to beta cells [42].

CFRD is a complication that affects approximately 50% of CF adults [43]. Even before the onset of diabetes, these CF children show impaired glucose tolerance. The primary risk factor for CFRD is exocrine pancreatic insufficiency [42]. Unlike Type I and Type II diabetes, CFRD exhibits features of both. It is mainly characterized by a loss of functional β-cell mass and insulin resistance.

#### 1.3.3. CF Gut

CF patients suffer from several GI issues, such as meconium ileus (MI), constipation, gastroesophageal reflux disease (GERD), and distal intestinal obstruction syndrome (DIOS) [44]. CF-associated constipation is experienced by almost 47% of CF patients. This number could be much higher, as CF-associated constipation remains underdiagnosed and underreported. Management of GI issues is important to improve the quality of life in CF patients [45]. MI is diagnosed in 10–20% of CF newborns [46]. In MI, there is a complete or partial bowel obstruction at the terminal ileum due to thick intestinal secretions [47]. Worsening of MI causes bowel wall distension and ischemia [48]. The usual course of treatment for meconium ileus includes nasogastric decompression, hyperosmolar enemas, intravenous hydration, and antibiotics [49]. This condition, if left untreated, could be fatal [45]. Furthermore, due to decreased bicarbonate production and gastric hypersecretions, CF patients have a propensity to develop peptic ulcers and duodenitis [50]. Meconium plug syndrome, intussusception, appendicitis, pneumatosis intestinalis, fibrosing colonopathy, bacterial overgrowth, and gastrointestinal malignancy could be observed in CF patients [35].

### 1.4. Cystic Fibrosis Transmembrane Conductance Regulator (CFTR) Gene

The CF gene, CFTR, was discovered in 1989 by positional cloning [51]. It was a landmark discovery in the CF field as it unfolded the opportunities to discover CF from the perspective of CFTR deficiency and narrow down the targets for CF therapy. 

The CFTR gene, which is approximately 250 kb in length, is located on the q31.2 position of the long arm of chromosome 7 and encodes for a 1480 amino acid long protein [52]. Mutations in the CFTR gene cause CF [53]. CF is predominantly observed in Caucasians, and the nature of the mutation determines the severity of the disease. Presently, more than 2000 CFTR allelic changes have been identified, and 719 of these mutations are known to be disease-causing. CFTR mutations can be classified into six broad categories, namely classes I–VI [54]. The classification is based on how these mutations affect CFTR protein synthesis, function, trafficking, and stability at the plasma membrane. Missense mutations (42%) are commonly observed in CFTR, but other mutations such as splicing (13%), frameshifts (15%), in-frame deletions, insertions (5%), and nonsense (10%) mutations have also been identified [55]. Classes I, II, and III mutations are known to cause classic CF phenotype associated with no residual CFTR function, and patients have severe phenotype. On the other hand, individuals with mutations belonging to classes IV, V, and VI normally present with a mild CF phenotype due to the presence of partial CFTR function [56,57,58].

The Class I mutation is due to a nonsense mutation (inserting a premature stop codon) that impairs CFTR protein synthesis [59]. The insertion of a premature stop codon activates nonsense-mediated decay, resulting in mRNA degradation. G542X, W1282X, and R1162X are some of the common mutations in this class [55]. Class II mutations affect CFTR protein processing, which impairs CFTR folding, trafficking, biosynthesis, and channel function. The misfolded protein becomes degraded via the ER-associated degradation (ERAD) pathway [60]. The most common CFTR F508del mutation is a Class II mutation [59]. Among CFTR mutations, the homozygous state of F508del accounts for 70% of the CF cases in the United States [1,2,61]. Most studies in the last few decades have been dedicated to understanding the complex behavior of the F508del CFTR protein and ways to fix it. Other candidate mutations in class II defects are R560T, A561E, R1066C, and N1303K [62,63,64,65]. The Class III mutation in CFTR leads to a gating defect in the CFTR channel. This mutation is usually a missense mutation located on the ATP binding domain of CFTR (NBD1, NBD2). A prominent example of a Class III defect is G551D CFTR [65,66]. Class IV mutation is a missense mutation that reduces CFTR channel function due to poor chloride ion conductance. R117H and R334W CFTR mutations belong to Class IV mutations [67]. The Class V mutation is known to have an impact on the quantity of functional CFTR protein. Generally, this is due to splicing mutations causing a significant reduction in the protein levels (e.g., 2789 + 5G → A, 3272 − 26A > G) [68]. Class VI mutations lead to reduced protein stability at the cell surface [69,70]. Post-ER and rescued F508del CFTR have considerably reduced cell surface retention [71]. Mutations in the C-terminal region of the CFTR protein can affect its maturity and membrane stability [70,72,73]. Some deletion mutations can remove the N-tail of the CFTR protein, which regulates its anchoring to the cytoskeleton, as seen in the case of c.120del23 [74,75,76]. Finally, some mutations typically cause impaired CFTR mRNA production, often due to large deletions in the CFTR gene (such as Dele2,3, 1717-1G → A), and have been subcategorized into Class I mutations [56,77,78].

Understanding the cause of defects in CFTR and the subsequent classification hold paramount importance in the development and identification of appropriate CF therapy. For instance, current FDA-approved CF drugs address the nature of CFTR defects and not a specific mutation [65].

### 1.5. Cystic Fibrosis Transmembrane Conductance Regulator (CFTR) Protein 

**Figure 2:**
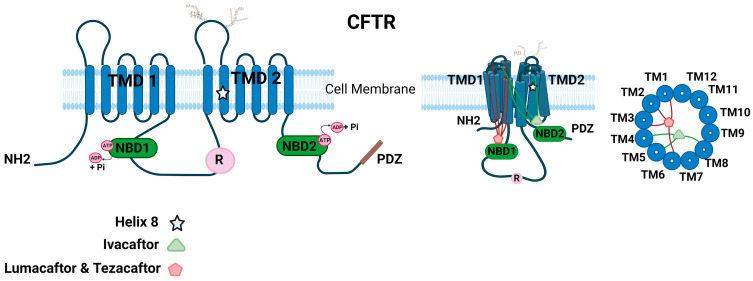
CFTR topology and structure: CFTR contains two transmembrane segments (TMD1 and TMD2); each TMD connects to cytoplasmic nucleotide-binding domains (NBDs) and a unique regulatory domain (R). Putative binding sites for some of the CFTR modulator compounds, ivacaftor, lumacaftor, and tezacaftor, are depicted based on the reported cryo-EM structures in the literature.

CFTR is a unique transporter in the ATP-binding cassette (ABC) superfamily of proteins that also functions like an ion channel [3,79]. CFTR contains an N-terminal lasso motif, two hydrophobic membrane-spanning domains, each consisting of six transmembrane helices, two cytoplasmic nucleotide-binding domains (NBD), and a unique~200 residue-long cytosolic regulatory domain (R) (Figure 2). The R domain consists of 19 predicted consensus phosphorylation sites for different protein kinases, such as protein kinase A (PKA), cGMP-dependent protein kinase II [cGKII], and protein kinase C (PKC) [80]. The amino and carboxyl ends facing the cytoplasm regulate key protein–protein interactions of CFTR [3,81]. CFTR contains a 32-amino acid (F^405^ to L^436^) largely disordered stretch in NBD1, known as the regulatory insertion (RI) region [82]. Deletion of the RI region enabled the rescue of F508del CFTR and restored Cl^−^ channel activity of CFTR as the protein could evade ERAD [82].

The CFTR channel activity is tightly regulated by kinases and phosphatases [83,84,85,86,87,88]. Among the various kinases, cAMP-dependent Protein Kinase A (PKA) is the key phosphorylation enzyme for CFTR [84]. The CFTR channel’s opening and closing are regulated by the addition or removal of phosphate residues. This modulation is primarily orchestrated through the phosphorylation of the R domain via PKA [83]. The CFTR Cl^−^ channel is open in the regulatory domain phosphorylated state when ATP binds, and this ATP binding facilitates NBD dimerization. On the other hand, ATP hydrolysis corresponds to the dephosphorylated and separated NBD state, leading to channel closure [89,90]. However, the phosphorylation mechanism through PKA is more intricate than the simplified version provided here. The channel’s access to PKA at the primary phosphorylation site determines the channel’s open probability, and PKA can either activate or inhibit CFTR gating. For instance, phosphoserines S422, S660, S795, and S813 activate CFTR gating, while S737 and S768 could either inhibit or activate CFTR gating [91]. In addition to enabling partial activation of CFTR, PKC is considered essential for the complete activation of CFTR channel function by mediating basal phosphorylation of serine residues 686 and 790 in the CFTR R domain prior to PKA-mediated phosphorylation [85]. Other kinases such as AMP-activated protein kinase (AMPK), Casein Kinase 2 (CK2), and p60Src tyrosine kinase have been reported to regulate CFTR by phosphorylation [86,87]. CFTR function could be regulated by interactions with many other proteins and ion channels that modulate the phosphorylation status of CFTR [81,92,93,94,95,96]. The C-terminal PSD-95/Discs-large/Zona occludens (PDZ) motif of CFTR is involved in many of these key regulatory interactions of CFTR [97,98]. 

### 1.6. Molecular Structure of CFTR Ion Channel

Given that there are several identified disease-causing mutations in CFTR, there is a critical need to understand the key regions that are essential for the structural and functional integrity of CFTR. Earlier attempts determined the high-resolution crystal structures for CFTR regions such as mouse NBD1 (mNBD1), which harbors the F508del mutation [13]. mNBD1 has a 78% identical sequence to human NBD1. The mNBD1 crystal structure was found to be unique compared to other ABC proteins. CFTR-NBD1 consists of regulatory segments, subdomain interconnection, and an unusual nucleotide conformation [13]. Conventional purification techniques and 2D crystalline arrays have resulted in low-resolution structures of recombinant human full-length CFTR and provided only limited information [99]. Finally, with the aid of electron cryomicroscopy (cryo-EM), a high-resolution structure of full-length human CFTR at 3.9 Å is now available [12]. Based on the human and zebrafish full-length CFTR structures, the ion conduction pathway consists of a large cytosolic vestibule, a narrow transmembrane (TM) tunnel, and a single gate near the extracellular surface. The large anion conduction pathway is lined by numerous positively charged residues [100]. The dephosphorylated regulatory domain is found inserted in the intracellular opening between the two cytosolic halves of the molecule and is positioned to prevent NBD dimerization [12]. Furthermore, as the R-domain is in contact with both halves of the molecule, it is predicted to substantially limit the conformational flexibility of CFTR, a feature at variance with other ABC transporters. A key proposition is that phosphorylation of the R domain by PKA is enabled by its infrequent spontaneous release events, which explains weak and rare residual ATPase as well as the gating activity of dephosphorylated CFTR [12,101]. Another proposition is that the binding of PKA to the R-domain rather than phosphorylation is critical for the extended release of the unphosphorylated R-domain from its occluded position [102]. The additional distinct feature of CFTR is the helix–loop transition in transmembrane helix 8, which creates a flexible hinge to facilitate gating and thus likely forms the structural basis for CFTR’s channel function. These studies provide key information to correlate structure to function and a likely explanation for the defects in many disease-causing mutations of CFTR. Another important structural feature of CFTR is an N-terminal interfacial lasso loop of about 60 residues that has functional significance, as underscored by several mutations in this region that cause CF [100]. The role of this motif has been described as mediating interaction with the membrane traffic machinery and regulating channel gating through inter-domain interactions [103,104].

### 1.7. Addressing the CFTR Protein Defect Using Small Molecules-CFTR Modulators

In the past decade, the median life expectancy of CF patients has significantly improved. CF patients’ life expectancy was one year or less when first described by Dorothy H. Andersen. Currently, the estimated age of survival of CF patients is close to 50 years [105].

The discovery of the gene CFTR about 30 years ago has paved the way for transformational care in CF today. Earlier, the management of CF disease was dependent on symptom-based treatments. These therapies improved quality of life and patient longevity and addressed symptoms such as pulmonary infection, inflammation, and mucus obstruction. Despite these advances, the median survival rate of people with CF remained low. In the past decade, several drugs that addressed CFTR defects were identified and are termed CFTR modulators. The CF modulators are small molecules that work on the defects in protein trafficking, gating, and synthesis of CFTR protein caused by specific CFTR mutations. Different categories of CF modulators include (i) Potentiators that help the CFTR channel to open [106]; (ii) correctors are pharmacologic chaperones that enhance CFTR trafficking to the plasma membrane by improving CFTR folding [107]; (iii) stabilizers are known to stabilize the CFTR variant at the plasma membrane [108]; (iv) amplifiers increase the amount of CFTR protein [109]; and (v) readthrough agents suppress the premature termination codons and help translate the full-length protein [67].

#### 1.7.1. CF Modulators in Clinical Application

CF modulators that gained regulatory approvals for clinical use are potentiators and correctors, all developed by Vertex Pharmaceuticals [59,110]. In 2012, the FDA approved ivacaftor (Kalydeco^®^), a CFTR potentiator for CF patients with the G551D CFTR mutation that has gating defects [111,112]. In 2015, the FDA authorized the use of Orkambi^®^, a combination drug consisting of lumacaftor (corrector) and ivacaftor (potentiator), for the treatment of patients homozygous for the F508del-CFTR mutation [111,113]. A study by Cholon et al. identified an unexpected detrimental effect of chronic treatment with ivacaftor on the lumacaftor-mediated correction of F508del-CFTR [114]. Symdeko, a combination of a next-generation corrector tezacaftor with fewer drug–drug interactions with ivacaftor, was later developed and approved in 2018 for patients with two copies of F508del-CFTR [111,115]. In 2019, the FDA approved Trikafta^®^, a triple combination therapy involving two correctors, tezacaftor and elexacaftor, and a single potentiator, ivacaftor, for patients who have at least one copy of F508del-CFTR [111,116]. The development of Trikafta^®^ has proven to be a game-changer in the field of CF treatment, with its remarkable efficacy and safety profile. 

CF patients with Class III and IV mutations who have channel gating and ion conductance defects could benefit from potentiators. Patients with G551D-CFTR mutation demonstrated dramatic improvement in their lung function from ivacaftor treatment with an improvement in their predicted FEV_1_ (Forced expiratory volume in 1 s) by 10.6 percentage points, and patients in ivacaftor group were 55% less likely to have a pulmonary exacerbation than patients receiving placebo [117]. The corrector and potentiator combination treatments from Orkambi^®^, Symdeko^®^, and Trikafta^®^ for patients who are homozygous for the F508del mutation or one F508del mutation and other responsive mutations have been shown to be safe and efficacious, resulting in significant improvement in lung function as well as a reduced rate of pulmonary exacerbation. In comparison to Trikafta, Orkambi and Symdeko have significantly lower efficacy [116,118,119,120]. While Orkambi and Symdeko have shown a reduction in the rate of pulmonary exacerbation and a modest improvement of FEV1 % pred between 3.4% and 6.7% compared to baseline [113], Trikafta has demonstrated a significant improvement in lung function of 10–14% of FEV1 % pred and respiratory-related quality of life for patients with one copy of the F508del mutation and minimal-function mutation, as well as patients with two F508del homozygous mutations [116,120]. This treatment has been life-changing for patients with the F508del minimal function genotype, for whom previous CFTR modulators were either ineffective or only moderately effective. Trikafta^®^’s clinical trials demonstrated a partial normalization of the mean baseline ppFEV1 (Precent predicted Forced expiratory volume in 1 s), ranging from 60% to 65%, after 24 weeks of treatment in F508del heterozygous patients [116]. Similarly, F508del homozygous patients showed partial normalization after only 4 weeks of treatment [120]. Several novel potentiators, such as VX-561, PTI-808, and ABBV-3067; novel correctors, like VX-121, PTI-801, and ABBV 2222; and an amplifier, PTI-418, are currently in trials and aim to further advance the efficacy of CF treatment [121]. 

Extensive efforts have been made to understand the mechanism of action of CFTR modulators. Knowing how potentiators and correctors act to relieve CFTR defects will provide a conceptual framework to understand the effect of small molecules on protein folding and their potential application to other misfolded proteins. The binding site for ivacaftor is now known based on the cryo-EM structure of ivacaftor in complex with phosphorylated E1371Q CFTR in the presence of saturating ATP-Mg^2+^ (10 mM) [122]. The ivacaftor docks into a cleft formed by transmembrane (TM) helices 4, 5, and 8. The predictable binding site is at the signature hinge region in TM 8 of CFTR. The extracellular region of TM 8 rotates around this hinge upon ATP binding that could be stabilized by ivacaftor. This study is critical for guided drug design to develop the most optimal therapeutic agents in CF.

Initial studies from our lab established direct binding of the corrector molecules to CFTR [123]. The precise binding sites are only recently known from the molecular structure of CFTR in a corrector-bound state [124]. Several clinically useful CFTR correctors have been discovered so far, all of which interact directly with CFTR through three distinct mechanisms of action [67,124,125,126,127,128,129]. These correctors are classified as type I, type II, and type III. It is expected that correctors targeting distinct structural defects of CFTR could work synergistically to rescue mutant protein expression and function at the plasma membrane. Type I correctors, such as lumacaftor (VX-809) and tezacaftor (VX-661), contain a 1,3-Benzodioxol-5-yl-Cyclopropane Carboxamide (BCC) headgroup and help in forming NBD1–MSD1 and NBD1–MSD2 interfaces. The type I correctors lumacaftor and tezacaftor bind at the same site in CFTR in both NBD-separated and dimerized configurations. There are data supporting the idea that VX-809/661 might bind to alternative sites, including the TMD/NBD1 interface and NBD1 [129,130]. The correctors occupy an internal cavity in TMD1 and link together four transmembrane helices 1, 2, 3, and 6 of CFTR, which are intrinsically thermodynamically unstable. Lumacaftor and tezacaftor do not alter the CFTR structure but rather stabilize TMD1 in its native conformation [124,128,129,131]. The pocket lining residues critical for lumacaftor and tezacaftor binding are R74, L195, A198, and S364, while another residue, K68, is specific for lumacaftor. The type I correctors can stabilize the partially folded TMD1, while inter-domain interactions relying on the synthesis of a full-length protein are impending. In this manner, CFTR, as it folds co-translationally, becomes less susceptible to degradation by protein quality control machinery. C18, ABBV/GLPG-2222, C3, and ARN23765 are other type I corrector molecules that share similar chemical structures and are likely to follow similar mechanisms of action [107,124,132,133]. ARN23765 is an attractive, highly potent CFTR corrector with a picomolar binding affinity, a high degree of synergy with other types of correctors, and no negative drug interaction with VX-770 [132]. Such high-affinity probes could additionally be valuable for CFTR structure–function studies. Type II correctors follow mechanisms distinct from type I correctors and could complement their action. They specifically target NBD2 and its interfaces to stabilize them. While currently there are no FDA-approved type II correctors, various research compounds have been discovered and are being studied [134]. Here are some examples of Type II correctors: VX-152, VX-440, Corr-4a, ABBV/GLPG-2737, and ABBV/GLPG-3221. The combination of different correctors can be used effectively to treat F508del and other type II CFTR mutations [135,136]. Type III correctors can either aid in NBD1 folding or hinder its unfolding. Examples of type III correctors include 4172, 3158, and elexacaftor (VX-445). VX-445, a type-III CFTR corrector, stabilizes NBD1 by binding at the interface of TH10/11 and the lasso motif [75,76,125,137]. This binding site of VX-445 indicates that it stabilizes ICL4 by keeping TH10/11 anchored to the lasso motif. Additionally, VX-445 counteracts lost intermolecular interactions between F508 and the aromatic pocket in ICL4.

#### 1.7.2. Other CFTR Modulators for Potential Clinical Application

An estimated 10% of CF patients do not benefit from FDA-approved modulators, either due to the nature of their CF mutation or a lack of evidence of the benefit of CF modulators for their CF [138]. Personalized therapies can help bridge the gap in therapy access for patients who could potentially benefit from CF modulators [139]. Additionally, ~11% of CF patients carry a nonsense mutation in at least one of the CFTR alleles, which results in the generation of a truncated CFTR protein that is not addressed by current modulator therapies [140,141]. Suppressing early translation termination caused by premature termination codons (PTCs), termed readthrough, is a potential treatment strategy for nonsense mutations in CF [142]. Tremendous efforts have been made to discover clinically useful readthrough agents. Nonsense suppression agents such as aminoglycosides have been shown to be unsuitable for clinical use because of their associated toxicity [143]. The safe agents discovered so far do not restore sufficient protein function to become an effective treatment for CF [144,145]. A clinically potent agent for CF patients with PTC mutations is currently an unmet challenge. ELX-02, [6′-(R)-Methyl-5-O-(5-amino-5,6-dideoxy-α-l-talofuranosyl)-paromamine sulfate, also referred to as NB124], a non-antibiotic aminoglycoside analog, was the latest to be evaluated for the treatment of CF in patients with at least one copy of G542X and other non-F508del mutations in phase II trials [146]. ELX-02 is a type of glycoside that selectively binds to eukaryotic ribosomes, aiding in the readthrough of premature stop codons. Through a series of cell- and animal-based experiments, including patient-derived organoids, ELX-02 demonstrated its effectiveness in restoring CFTR function and synergistic improvement in CFTR function when combined with ivacaftor and the corrector’s lumacaftor and elexacaftor. However, the phase 2 study did not achieve statistical significance in clinical efficacy for ELX-02, including changes in sweat chloride concentration (SCC) and percent forced expiratory volume (FEV1) [Eloxx Pharma reports phase 2 results for the ELX-02 combo trial in class 1 CF patients; source: 22 September 2022, Eloxx Pharmaceuticals]. Additionally, there was no improvement in the effectiveness of the treatment, even in combination with ivacaftor. 

There is a substantial degradation of the CFTR RNA-bearing nonsense mutation due to nonsense-mediated mRNA decay (NMD), and the RNA level could be as low as 1.7% of WT [147]. Therefore, there is a proposition to first prevent this substantial RNA decay before efforts to improve CFTR protein function could be successful. A rationalized consensus on the effective rescue of CFTR with nonsense mutations is to (i) promote PTC readthrough, (ii) inhibit NMD, and (iii) apply CF modulators to enhance the function of the resulting CFTR protein [148]. Newly identified eukaryotic release factor 3a (eRF3a) degraders CC-90009 and SJ6986 promote PTC readthrough for nonsense mutations by targeting cereblon E3 ligase, which induces proteasomal degradation of eRF3a and eRF3b, the proteins that facilitate translation termination [149]. Treatment with CC-9009 led to robust CFTR rescue in nasal and bronchial cells with the W1282X/W1282X mutation, an effect that could be further enhanced using CF modulators. This compound also resulted in a significant reduction in the activity of the epithelial sodium channel (ENaC). Additionally, ERF3a degraders synergize with aminoglycosides to further boost CFTR rescue. SRI-37240 and its derivative, SRI-41315, are other investigational drugs that have been shown to suppress *CFTR* nonsense mutations [144]. These agents reduce the levels of termination factor eRF1, which promotes readthrough and enhances mRNA abundance. However, SRI-37240 and SRI-41315 only showed modest improvements in CFTR currents, and the significant improvement was mostly in synergy with aminoglycoside G418.

Several other potent modulators for common CF mutations have been developed and tested. Pedemonte et al. earlier identified phenylglycine, tetrahydrobenzothiophene, and sulfonamide potentiators to correct F508del CFTR channel gating by increasing the channel opening time [150]. The use of these potentiators proved to be successful in human CF airway epithelial cells that express F508del-CFTR. These potentiators, by themselves or in combination with CF correctors, can be used as a therapeutic approach to treat CF patients caused by the F508del mutation [150]. A new type of CFTR corrector has been discovered, called tricyclic pyrrolo-quinolines, which has proven effective in primary airway epithelial cells from CF patients with the F508del mutation through a functional test [151]. PP028 is the most effective compound within this family of correctors. When paired with VX-809 and VX-661, it has shown synergy, but not with VX-445. Amino acids 1-387, which are part of the amino-terminal portion of the protein, are where VX-809 has been found to be most effective. On the other hand, PP028 and VX-445 [151] have a stabilizing effect only when amino acids 1-1181, which include the second membrane-spanning domain, are included. Tricyclic pyrrolo-quinolines, similar to VX-445, could be a promising option for correcting CFTR and may be used in combination with other pharmacological treatments to address the fundamental defect in CF [151].

Other AbbVie/Galapagos compounds targeting rare mutations, including AC1, AC2-1, and AC2-2, have therapeutic potential [135]. A combination of AC1, AC2-1, and the potentiator AP2 proved efficient in correcting the defects of M1101K- and G85E-CFTR proteins in nasal cells taken from patients. The response to this novel combination was significant in cells expressing M1101K-CFTR, ranging between 100 and 280% of the average forskolin response in non-CF cultures [152,153].

### 1.8. Non-CFTR Therapies in CF

Many pharmaceutical companies are showing an increased interest in developing additional therapies for CF, not just by designing new CFTR modulators but also by exploring alternative molecules that could indirectly improve CFTR function. It is important to note that CFTR is not the only target for therapies aimed at restoring epithelial homeostasis in CF. Other small molecules targeting non-CFTR channels, like ENaC and TMEM16A, are also being developed and show potential for patients who are not responding to already-approved CF therapeutics. The ENaC inhibitor BI 1265162 is currently in phase 2 clinical trials and has shown promise for optimizing outcomes for CF patients, regardless of their CFTR mutation class [154]. Continued research in this direction is expected to bring new CF therapies that could be used alone or in combination with approved modulators to improve outcomes for currently ineligible patients with rare CFTR mutations [155].

### 1.9. Prospective Gene Modification Therapies in CF 

The current limitation of CFTR modulators is that they only work for specific mutations, leaving around 10% of people with rare genotypes without effective treatment. As a result, there is a growing interest in developing alternative treatment approaches that are applicable to CF patients irrespective of their mutation type. These strategies may include genetic supplementation or editing, such as delivering DNA or mRNA that encodes for normal CFTR or restoring the normal CFTR gene through genome editing or mRNA editing [156].

Gene therapy in CF is currently aimed at replacing the faulty CFTR gene with functional copies in the epithelial cell layer of the airways, but there are several limitations to this approach. Choosing the right plasmid DNA molecule model is crucial for clinical potency, and delivering genetic therapies to the lungs is challenging due to various in situ barriers such as thick mucus, immune responses, and intracellular uptake [157]. Additionally, since the airway epithelium is continuously renewing, repeated administration is required, making it important to select the appropriate delivery method. Viral vectors such as adenoviruses [158,159,160], lentiviruses, and adeno-associated viruses [161,162,163] are the most common agents that are tested for CF gene therapy, along with peptide nanoparticles and non-viral lipoplexes [161,164,165]. Despite evidence of CFTR expression, many trials have not achieved clinical efficacy [159,160,161,162,166].

mRNA-Mediated Therapy: Research studies have found that chemically modified WT CFTR (cmCFTR) mRNA has been successful in restoring CFTR currents in CFBE41o-cells with the F508del mutation [167]. Robinson et al. conducted a study that demonstrated the effectiveness of lipid-based nanoparticles (LNPs) for delivering cmCFTR mRNA to bronchial epithelial cells in a clinically relevant setting [168]. This approach led to a significant increase in CFTR levels that were specifically localized to the membrane, consequently restoring its chloride channel function. Notably, LNP-cmCFTR administered nasally was able to recover CFTR-mediated Cl- secretion in CFTR knockout mice for a period of at least 14 days [168]. 

Artificial RNA-like molecules known as antisense oligonucleotides (ASOs) can selectively modify gene expression by correcting abnormal mRNA or targeting RNA transcripts for degradation [169]. In the context of CF, ASOs have shown promise for improving CFTR function and reducing lung symptoms. For instance, the modified RNA oligonucleotide QR-010 has been found to enhance CFTR function in the nasal mucosa of patients with the F508del mutation [170]. Additionally, ASOs have been found to modulate CFTR function in cells with CFTR splicing mutations [171]. One example of a common mutation in the CFTR gene is CFTR c.3718-2477C>T, which generates a 5′ splice site leading to the generation of an 84 nucleotide pseudo-exon with a premature termination codon [172]. By using 2′-OMe/PS splice-switching ASOs along with oligonucleotide-enhancing compounds, it is possible to target the pseudo-exon and restore the normal splicing pattern of CFTR mRNA [173,174]. Studies conducted on CF patient-derived HBE have shown that the use of splice-switching ASOs, along with CFTR modulators, has improved CFTR function compared to the use of CFTR modulators alone [175,176]. As an alternative to targeting CFTR, recent studies using mouse models have demonstrated that inhalable ENaC-specific ASOs can downregulate ENAC and mucus expression and alleviate goblet cell metaplasia, airway hyper-responsiveness, and inflammation [177]. 

The CRISPR/Cas9 technology is a breakthrough discovery that holds the potential for treating genetic diseases such as CF. Schwank et al. conducted pioneering studies using CRISPR/Cas9 to correct the F508del mutation in the CFTR gene, resulting in improved functionality of the CFTR protein in intestinal organoids derived from CF patients [178]. They proposed the transplantation of corrected organoids as a possible treatment approach. Additional studies by Firth et al. and Crane et al. utilized CRISPR technology to correct CFTR gene mutations in iPSCs, demonstrating restoration of CFTR function in lung epithelial cells [179,180]. Sanz et al. explored CRISPR/Cas9-based methods to edit specific CF-causing mutations: c.3140-26A>G, c.1679+1634A>G, and c.3718-2477C>T CF, achieving successful excision through the non-homologous end joining (NHEJ) pathway in a significant proportion of transfected cells [181]. These studies collectively highlight the potential of CRISPR/Cas9 technology in developing targeted therapies for CF by correcting genetic mutations associated with the disease.

Recent discoveries in stem cell therapy have shown that induced pluripotent stem cells (iPSCs) hold promise for effectively repairing tissue defects. They are patient-specific, non-immunogenic, and easily modifiable [182]. The initial discovery of iPSCs was made by Yamanaka’s group, which reprogrammed mouse fibroblasts and subsequently confirmed the results using human fibroblasts [183]. It has been demonstrated that CRISPR/Cas9 gene editing techniques can repair a CFTR mutation in humans [180]. These repaired stem cells from individuals with CF could be reprogrammed into lung niches and subsequently differentiated into respiratory cells [179,184]. Moreover, CRISPR/Cas9 has demonstrated the full recovery of CFTR protein function in organoids derived from CF patients [178]. iPSC assays, when combined with CRISPR/Cas9 gene editing techniques, could become a powerful approach for stem cell-based regenerative therapies. The creation of a range of iPSC lineages with the characteristics of different CF mutations would provide an effective means of selecting potential drugs to repair functional deficiencies [185,186].

### 1.10. CFTR Expression Defined Cellular Compositions in the Lung

The lung is a vital organ consisting of a progressively branching network of airways that close into terminal alveoli. Using single-cell transcriptomics, researchers have now charted the cellular landscape of the upper and lower lung airways. The major epithelial clusters in the human respiratory tree correspond to basal, ciliated, club, goblet, and alveolar cells [187,188]. The rare cell types include tuft cells, neuroendocrine cells, and ionocytes. Basal cells, which are the stem cells of the airway epithelium, serve as the main reservoir of rare cell types [187]. Goblet and ciliated cells are primarily derived from club cells [189]. 

The airway submucosal glands (SMGs) are located below the superficial respiratory epithelium and are made up of many branching secretory tubules. These tubules combine to form the main collecting duct, which leads to the ciliated duct and, eventually, the airway surface. The SMGs are primarily composed of mucous and serous cells and have their own distinct transcriptional profiles that differ from the secretory cells found in the respiratory epithelium [190,191,192]. The knowledge of the differences in CF and non-CF SMGs for cell-type-specific contribution is limited. A recent study in the CF porcine model suggests that normal and CF SMGs are structurally very similar, and the associated cell clusters do not show any major transcriptional differences, suggesting that the defects are primarily from the loss of ion transport activity from CFTR [193].

As the lungs near the end, the alveoli consist of the pulmonary alveolar epithelium, which is predominantly composed of two types of epithelial cells known as alveolar type I (AT1) and type II (AT2) cells [194]. The different airway epithelial subtypes have unique functions and gene expression profiles. Many respiratory diseases, such as cystic fibrosis (CF), asthma, and chronic obstructive pulmonary disease (COPD), may begin in the small airways during the early stages of the disease, which could be explained by the changes in cell composition [195,196,197,198]. Therefore, it is critical to understand the single-cell dynamics of the lung from the perspective of CFTR to understand the disease pathology and for precision medicine in CF. The epithelial sub-types are discussed in the order of the representation of CFTR expression per cell: ionocytes > secretory > basal cells >> ciliated cells. 

#### 1.10.1. Ionocytes

Resolving the question of which cell types within the human lung express CFTR is required to devise molecular strategies for CFTR gene correction. Ionocytes were identified as cells with distinctively high expression of CFTR among the epithelial cell sub-types [199,200]. However, they are quite rare, account for <5% of all *CFTR*^+^ cells, and are more abundant in the tracheobronchial regions (large airways) [201,202]. The transcription factor known as forkhead box I1 (FOXI1) plays a crucial role in the specification of ionocyte progenitors in multiple species [199,200]. *Foxi1*-knockout (*Foxi1*-KO) mouse airway epithelia have decreased *Cftr* expression; however, they show paradoxical forskolin-responsive anion transport, alluding to the likely compensatory mechanisms supported by other cell types and/or channels in mice that are different from other species such as humans, ferrets, and pigs [200]. CF mice do not spontaneously develop lung disease, unlike CF humans, pigs, and ferrets [203,204]. For this reason, a recent investigation into the ferret was critical to studying pulmonary ionocyte function [205]. *FOXI1* KO ferret’s airway cultures displayed reduced CFTR-mediated Cl**^−^** and HCO_3_^−^ currents by 69% and 68%, respectively, and significantly perturbed airway fluid properties compared with the wild-type [205]. Based on consensus gene expression profiling of 449 pulmonary ionocytes from both wild-type and *FOXI1*-Cre^ERT2^::ROSA-TG ferret airway ALI cultures, it was discovered that ferret pulmonary ionocytes were specifically enriched in four ATP6V0 and nine ATP6V1 family proton pumps, as well as chloride channels (*CFTR* and *CLCNKA*), NKCC1 basolateral Na-K-Cl symporter (*SLC12A2*), *BSND* (activator subunit of *CLCNKA)*, ENaC channel α and γ subunits (*SCNN1A* and *SCNN1G*), potassium channels (*KCNQ1*, *KCNK1*, *KCNMA1*, and *KCNK5*), and the ATPase Na^+^/K^+^ transporting channel (*ATP1B1*), consistent with the high ion transport capability in this subpopulation. This study also identified sub-types of ionocytes based on single-cell gene annotation and the presence of distinct transcriptional programs. Type A pulmonary ionocytes are enriched for known ion/osmoregulatory roles of ionocytes in other systems. The type B ionocyte is distinguished by a distinct set of enriched genes pertaining to IL-8 signaling, inflammatory response, and infectious disease pathways. Aquaporin gene expression (*AQP3*, *4,* and *5*) is notably abundant in subtype C, suggesting their possible unique function in water transport. Type C ionocytes appear to share a close lineage with rare cell progenitors that specify tuft cells, PNECs, and ionocytes, thus making them committed progenitors of both type A and B ionocytes. Importantly, the transcriptional signature of human pulmonary ionocytes was significantly more similar to that of ferrets than to mouse ionocytes, underscoring the significance of the ferret model in studying CF in humans.

Strategies to enrich the ionocyte population have been adopted using AAV-assisted expression of FOXI1, which was shown to increase the ionocyte population by 7-fold and total CFTR mRNA and protein levels by 10-fold and 2.5-fold, respectively [206]. Ionocyte enrichment resulted in elevated basal currents for CFTR [206]. Sonic hedgehog (SHH) signaling could be an essential signaling cue for human basal cell specification of ionocytes [207]. Activation of the SHH pathway with the chemical agonist SAG significantly enhanced the abundance of CFTR^+^ BSND^+^ ionocytes, which correlated with an increase in CFTR-mediated currents in differentiated airway cultures. 

Pulmonary ionocytes can be derived from human induced pluripotent stem cells (iPSCs) through directed differentiation during development [208]. iPSC-derived airway basal-like cells (iBCs) that exhibit the functional features of tissue-resident airway stem cells can specify ionocytes in addition to the more common trilineage airway epithelial differentiation repertoire. The iPSC-derived putative ionocytes were present at ∼1% frequency, which is consistent with the published frequencies of primary ionocytes in human and mouse large airways. iPSC-derived ionocytes expressed most CFTR per cell compared with other cell types but contributed to only 7.5% of total CFTR^+^ cells. Consistent across various studies, ionocytes express the highest level of CFTR but form a minor proportion of total CFTR^+^ cells. All these data have utility in defining the path for precise genome editing for the treatment initiatives in CF. 

#### 1.10.2. Secretory Cells

The secretory cells are abundantly present in the airway epithelium and play a crucial role in producing and secreting mucus and other protective substances [20]. Secretory cells comprise the mucous, goblet, club cells, and serous cells of the submucosal gland acini [209]. Mucous cells on the airway surface produce and secrete protective mucins, primarily MUC5AC and MUC5B, while mucous cells of submucosal glands are MUC5AC^−^/MUC5B^+^ [210]. Club cells secrete antimicrobial and anti-inflammatory peptides involved in the host defense mechanism, including Clara cell secretory protein (CCSP) and secretoglobulin (SCGB) family members. Serous cells secrete electrolytes, water, and various antimicrobial proteins that later mix with the mucins secreted from the mucous tubules and then into a collecting duct and onto the airway surfaces [193,211]. Large airway secretory cells are marked by *SCGBA1,* while small airway cells express *SFTPB* and *SCGB3A2.* The human respiratory bronchioles also contain distinct secretory cells termed respiratory airway secretory cells (RAS; marker genes: *SCGB3A2*, *KLK11*, *SOX4*) that act as unidirectional progenitors for AT2 cells, which are essential for maintaining and regenerating the alveolar niche [212,213]. 

Recent studies using scRNA-seq have identified sub-clusters of secretory cells with unique gene expression profiles and functional roles that are now known to have differences between normal and CF lungs [214]. CF lung samples demonstrated significant enrichment of antimicrobial programs in the serous- and mucous-like subsets, suggesting that CF lungs develop specialized antimicrobial activity that is expected in response to the infection [214]. Goblet-like cells in CF lungs showed high levels of ER stress, and both goblet- and club-like cells displayed more pronounced metabolic differences from the control samples [214]. 

Although CFTR is overrepresented in ionocytes, secretory cells are the most common cell type expressing *CFTR*, followed by basal cells, which together account for approximately 80% of *CFTR*^+^ cells. Ionocytes account for <5% of all *CFTR*^+^ cells and are more abundant in the tracheobronchial regions (large airways) [202]. The total expression of CFTR in the secretory cells correlates with their highest contribution to CFTR function in the small and large airway epithelial cells. The iPSC-derived differentiated airway cultures also showed consistency with the primary airways, with secretory cells constituting the majority of all *CFTR^+^* iPSC-derived airway epithelia at 63%, while 20% were basal secretory transition cells, 3.9% were basal cells, 4% were immature ciliated cells, and 1% were mature ciliated cells [208]. Ionocytes, as described earlier, accounted for 7.5% of the total *CFTR^+^* cells. Secretory cells are also the primary *CFTR^+^* responders to the purinergic stimuli (ATP and UTP) to regulate ASL [202].

#### 1.10.3. Basal Cells

Major portions of the airway epithelium are pseudostratified. Basal cells are the airway tissue stem cells that are situated next to the basement membrane and contact luminal cell types, including ciliated cells and various secretory cell types [199,215]. Different basal cell subsets are identified by canonical basal cell markers, including *TP63* (tumor protein P63), *KRT5*, and *KRT15* (cytokeratins 5 and 15) [199]. Basal subsets can be highly proliferative self-renewing cells or cells transitioning to a secretory phenotype. 

Differential gene expression analysis between the control and CF samples revealed a significant reduction in the abundance of cycling basal cells [214]. CF basal cells were overall identified as having high metabolic fragility, low proliferative capacity, and epithelial functions. The defects in CF basal cells have serious implications for the proficiency of airway repair and remodeling in CF. It is known that basal cells are dynamically regulated in response to disease and infection and become biased toward certain lineages in disease-specific contexts, such as basal to ciliated in CF and basal to secretory in idiopathic pulmonary fibrosis (IPF) [214,215,216]. Modulation of basal cell plasticity is an attractive approach toward restoring the equilibrium of perturbed airway epithelial states associated with CF. 

#### 1.10.4. Ciliated Cells

The ciliated cells are readily identifiable by the presence of microtubular protrusions called cilia. Ciliated cells are highly abundant cell types and continue to increase as airways branch more (47 ± 2% in the trachea to 73 ± 1% in the small airway epithelium) [30]. Cilia provide the mechanical force to expel mucus, debris, and infection from the lungs, which, together with mucus, is part of the mucociliary escalator. The expression of CFTR in ciliated cells, which form a dominant airway sub-type in native and ALI airway cultures, is now largely disputed and is known to be low and infrequent [202,212]. Despite their abundance, ciliated cells account for only 5.6% of total *CFTR*^+^ cells [202]. The earlier reported evidence of CFTR expression in the apical pole of ciliated cells in human bronchial epithelium is suggested to be anomalous due to the nonspecific reactivity of commonly used CFTR antibodies to a ciliary protein [217].

Ciliated cells were also found to be altered in CF compared to the control samples, with a significantly higher ciliogenesis signature in CF cells [214]. This coincided with an increased rate of direct basal to ciliated cell differentiation in CF cells that was concluded based on the significantly increased expression of markers for early ciliogenesis in basal cell subsets. This was further validated by in situ hybridization, confirming the presence of cells with dual expression of *KRT5* and *LRRC6*. These cells were located in the suprabasal position, which is consistent with their transitional phenotype from the basal cell hub to the airway lumen, and were significantly enriched in CF. The authors further emphasized the overrepresentation of the ciliogenesis signature that is marked by *FOXN4* in CF and represents transitional *FOXJ1*^+^ cells undergoing multiciliogenesis.

Many respiratory conditions, such as chronic obstructive pulmonary disorder (COPD), non-CF bronchiectasis, primary ciliary dyskinesia, COVID-19, asthma, and IPF, have underlying defects in CFTR function or expression and share many pathological features of CF (molecular, architectural, and functional) [29,218,219]. Through a highly resolved study approach on CF using analytical tools for single cells, we might be able to navigate many undefined and unconventional roles of CFTR in human lung physiology. This remains important as we are still far behind the goal of finding a complete cure for CF. Given that the concepts emerging from single-cell studies are redefining how we understand a disease, it is essential to carry out corroborative studies along this route, e.g., from transcripts to function to disease at a single-cell resolution, and faithfully capture the dynamics of the interacting communities of cells and microenvironment in the naïve and diseased states [220,221]. Transcriptional profiling of immune cells in spontaneously expectorated CF sputum has reconfirmed specific signatures of immune dysfunction, i.e., the airway immune cell repertoire shifts from alveolar macrophages in healthy individuals to recruited neutrophils and monocytes in CF patients [222]. Based on transcriptional segregation, there are three types of mononuclear phagocytes that are abundant in CF: activated monocytes, heat shock-activated monocytes, and monocyte-derived macrophages. Neutrophils, being the most abundant type of immune cell in CF, exhibit a dominantly immature and pro-inflammatory profile. CF monocytes and neutrophils, although highly pro-inflammatory, showed maladaptive programs for phagocytosis and cell survival. Single-cell studies have already been conducted in the other types of epithelia in addition to the respiratory tract, including the gastrointestinal tract, pancreas, and urogenital tract, and have defined specialized populations for CFTR that need to be investigated in the context of CF disease in these organ systems [223,224,225].

### 1.11. Altered Protein Interactions Determine the Etiological Course of CFTR-Related Disorders

**Figure 3:**
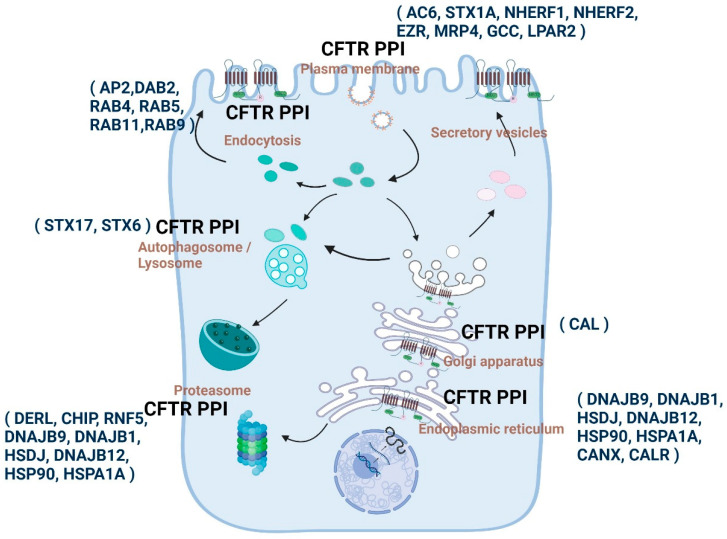
Sub-cellular PPI domains of CFTR, along with a few CFTR interactors in each domain based on the literature and our own experimental findings.

The regulation of CFTR is dependent on protein–protein interactions (PPIs). A number of proteins have been identified that interact with CFTR in the cellular space and context of time (Figure 3) [152,153]. Many CFTR interacting proteins bind through the amino and carboxyl ends of CFTR [81]. CFTR PPIs play essential roles in protein folding, trafficking, membrane localization and recycling, and degradation and are highly specialized for sub-cellular compartments [154,155]. The dynamic nature of these interactions enables the titration and fine-tuning of CFTR-dependent physiological responses to various environmental stimuli [156]. Consistently, disruptions in CFTR-PPIs, especially for the quality control mechanisms, have pathological consequences that are known to be associated with CF [157,158]. 

To advance treatments for cystic fibrosis (CF), it is necessary to have a thorough understanding of the composition of proteins that are associated with both normal and mutant CFTR. Due to the highly effective CFTR modulator therapies that are available to >90% of the CF population, it becomes imperative to comprehend these interactions in the context of the modulators and how they may impact the short-term and long-term benefits of the treatment.

#### 1.11.1. Snapshot of the Molecular Communication between CFTR PPIs and CF by Various Proteomic Approaches

Through a unique application of the bio-orthogonal click chemistry approach that was much earlier than the discovery of cryo-EM structures of modulator-conjugated CFTR, Sinha et al. provided evidence for direct interaction between VX-809 derivatives (that are suitable for click chemistry reactions) and CFTR [160]. One of the key observations in this study was the significant accumulation of CFTR-VX-809 derivatives-conjugates in the peri-nuclear/ER regions, suggesting that the drug binds the mutant CFTR while the protein is being synthesized, as corroborated by several subsequent studies [124,130,226,227,228]. We also know that VX-809, based on its action on mutant CFTR folding, initiates, breaks, or reinforces many sub-compartmental interactions of CFTR while facilitating trafficking and further stabilizing CFTR at the plasma membrane through peripheral interactions with NHERF1 and the cytoskeleton-linker protein Ezrin and other unknown mechanisms [229]. Studies from our group have shown that the F508del CFTR binds poorly with NHERF1 compared to the WT CFTR. However, the presence of VX-809 enhances the binding affinity between CFTR and NHERF1, leading to the stabilization of F508del-CFTR at the plasma membrane [229].

First, a comprehensive analysis of the CFTR proteome came from the studies by the Yates group. They developed a more efficient method for CFTR immunoprecipitation, Co-Purifying Protein Identification Technology (CoPIT), to profile protein–protein interactions across the samples of HBE41o—(WT CFTR) and CFBE41o—(F508del CFTR) bronchial epithelial cell lines [230]. A total of 576 and 430 protein high-confidence interactors of F508del CFTR and WT CFTR, respectively, were identified, with an 85% overlap of the interacting proteins. Major changes were associated with the biogenesis of WT vs. mutant CFTR. Increased recruitment of chaperones, such as Hsp90, along with augmented protein degradation of F508del CFTR, is facilitated by a protein network that significantly differed from that of the wild-type CFTR and included up to 25% of the F508del CFTR-specific interactions. Significant protein interaction alterations were observed in the cells with the F508del CFTR mutation, affecting various processes such as translational control, protein insertion into the ER, protein transport, trafficking, N-glycosylation, anchoring at the plasma membrane, mRNA decay, and endocytic recycling. These changes indicate that F508del CFTR has a profound effect on protein biogenesis. For example, F508del CFTR is uniquely associated with the ER quality control and sugar transferase UGGT for re-glucosylation and eventual association with ERAD components. WT CFTR is associated strongly with the components of Wnt and mTOR signaling pathways, while F508del CFTR largely involved proteins in TGF-beta and JAK/STAT signaling. 

A follow-up study investigated the post-translational modification (PTM) code for WT vs. F508del CFTR [231]. This study identified 37 distinct PTM sites in either wild-type or F508del CFTR, consisting of 20 phosphorylation, 11 ubiquitination, and 6 methylation sites. Methylation sites are enriched in WT CFTR, while, as expected, ubiquitination sites were detectable only in F508del CFTR. Earlier phosphorylation of CFTR was only viewed as the primary PTM for regulating CFTR activity. In this study, it was demonstrated that phosphorylation of Thr421, Ser427, and Ser422 in the regulatory insertion (RI) element of CFTR is essential for proper CFTR biogenesis. RI phosphorylation is deemed essential for protein glycosylation and preventing the ERAD cascade by restricting the association of CFTR with ubiquitination machinery. An important takeaway observation from this study is that methylation–phosphorylation hotspots are highly dynamic for F508del CFTR, and the changes in phosphorylation sites and amounts upon misfolding are coincidental with the replacement of adjacent methylation with ubiquitination sites. Methylation to ubiquitination shift that occurs at many sites for F508del CFTR, such as for the ER exit motif, might signal that the protein is misfolded, preventing its ER exit and routing it for ERAD. These data suggest that F508del CFTR-altered proteome and PTM code may reflect the molecular route for disease pathogenesis and should be investigated in the context of primary tissues and disease microenvironment. 

The newly emerged revolutionary technique of proximity labeling (PL) has overcome the limitations of traditional strategies of investigating PPI in their native context. PL is a technique to perform site-specific proteomic mapping in living cells [232]. PL relies on the ability of certain engineered enzymes (peroxidase or biotin ligase-based) to convert an inactive small-molecule substrate into a highly reactive and transient diffusible intermediate that labels endogenous biomolecules within a small radius ≤ 10 nm in a promiscuous manner [233]. The major advantage of PL is that the labeling of biomolecules occurs in their native states while the spatial relationships and interaction networks are maintained. Covalently modified proteins can then be selectively enriched (e.g., with streptavidin-conjugated beads) for subsequent identification of the labeled molecules using mass spectrometry. A new generation of biotin ligase TurboID is available that has >100-fold faster labeling kinetics and much superior labeling yields than the original BioID [234]. TurboID-based proteomic labeling only requires biotin addition without any need for cofactors or co-oxidants. Recently, Dr. Ting’s group applied the complementation approach to achieve greater target specificity, in which TurboID was split at L73/G74 (split-TurboID) to generate two TurboID fragments (Tb(N), N-terminal fragment, and Tb(C), a C-terminal fragment), which can be reconstituted into a functional enzyme when brought together by a protein–protein interaction or membrane–membrane apposition [233]. Using the PL approach, information on the global and sub-compartmental states of the human CFTR interactome has begun to unravel [235]. Gene ontology enrichment analysis performed using proximity labeling showed an abundance of SNAREs and Rab small GTPases in the PPI complex, while the CFTR protein is largely incorporated into intracellular trafficking vesicles and navigates its path to the plasma membrane [235]. The PPI complex for CFTR includes chaperones and proteins involved in the ubiquitination process that are largely operative and aberrant in the context of mutant CFTR, as earlier elucidated [230,231]. Toward this, the CFTR-WT interactome was compared to that of the mutant forms of CFTR (G551D and W1282X mutations), which determined that while the G551 CFTR largely retains the PPI profile for WT-CFTR, there was a significant proteomic shift for W1282X CFTR, e.g., high association with a protein involved in misfolded protein response, and several of the NHERF-mediated interactions become highly perturbed for W1282X CFTR, which is to be expected because of the absence of the C-terminal PDZ motif [235]. However, in this study, the protein expression of W1282X CFTR was not clearly indicated, as this mutation significantly impairs the expression of CFTR due to the high rate of NMD. Although the PI approach offers many advantages over the conventional co-immunoprecipitation co-IP approaches, combining different approaches in different cell types might still be necessary to obtain a comprehensive picture of the CFTR interactome. PL will identify both transient partners and non-interacting but proximal proteins, which might not be co-immunoprecipitated, and several of the cell-specific differences have been observed in terms of the composition of the CFTR interactome [230,236]. A Proteome Variant Approach (ProVarA) was adopted to gain insights into the emergence and progression of variant-specific diseases such as CF [236]. This study provided important insight into the common (new and pre-validated) CFTR-PPIs that could be exploited to target different variants across a single class of CFTR mutation as well as the PPIs that are variant-specific. For example, pre-validated targets such as PDIA4, LGALS3BP, and PTBP1 were detected, and new targets such as PDIA1 and UNC45A, chaperone proteins that have a known regulatory function in protein folding, were additionally identified for the functional correction of the F508del variant. At the same time, none of the other corrector targets for ER-restricted variants included HspA5, calmodulin, PDIA1, LMO7, and PGRMC1, while HspA8, PDIA4, LGALS3BP, PTBP1, RPS2, and RPS6 demonstrated high selectivity toward the F508del variant. The effect of FDA-approved therapeutics was also shown to mediate pro-corrective changes in some of these PPIs and shift the PPI landscape toward WT-CFTR PPIs. ProVarA could be a robust approach to ascertain the undefined trajectory of PPIs driving disease pathogenesis and devise variant-specific therapies for non-responders to CFTR modulators and currently ineligible patients. 

#### 1.11.2. Cellular Sub-Compartmental State and Composition of CFTR PPIs and PPI Enabled Highly Compartmentalized CFTR Signalosomes

Proteins that interact with CFTR in different cellular compartments can be broadly categorized according to the different stages of CFTR biogenesis and its final delivery to the plasma membrane. The compartments include the nucleus [237], cytoskeleton [238,239], ER [230,240,241], Golgi apparatus [240,241,242,243], membrane trafficking vesicles [244], proteasome [67,230,241,242] and plasma membrane [243,244,245]. The composition of CFTR PPIs in different compartments differs significantly between WT-CFTR and CFTR mutants [230,236,246,247]. 

During intracellular CFTR trafficking, protein folding undergoes complex co- and post-translational processes [248]. CFTR folding begins during translation, and the early steps of folding are carried out in a modular manner (domain by domain) through domain–domain interaction, allowing its native conformation and insertion of the transmembrane segments into the ER. A signal sequence on the transmembrane is important for defining the CFTR topology and folding at the ER membrane [249]. The signal sequence is recognized by the signal recognition particle as the transmembrane emerges from the ribosome, which helps in the insertion of the transmembrane segments into the ER through the Sec61 translocon and positions the CFTR protein in the right orientation in concert with post-translational folding [250]. The primary steps in CFTR folding are the insertion of the TMD1 domain into the ER membrane, followed by the synthesis of NBD1 [248]. NBD1 interacts with Hdj2 and Hsc70 chaperones that regulate its folding and stabilization during R-domain synthesis [251]. Synthesis and insertion of the TMD2 domain occur after NBD1 and R domains emerge and fold into the cytosol by a co-translational mechanism [252], along with the release of chaperones. The final step is represented by TMD1-TMD2 complex formation and NBD2 domain synthesis, followed by post-translational maturation of CFTR conformation [253,254]. After the CFTR protein is properly folded and oriented, it is transported to the Golgi apparatus, where it undergoes glycosylation and maturation. The mature CFTR channel is finally transported to the plasma membrane through secretory vesicles [255]. CFTR is subject to quality control, whereas domain assembly and folding processes are prone to errors, regulating the properly folded proteins to reach the plasma membrane [255]. Some of the misfolded proteins are recognized and degraded by ER-associated degradation (ERAD) [255]. The assessment of quality is dependent on important protein interactions that occur either during the initial secretory pathway at the ER or later in the late secretory pathway at the Golgi, PM, and endosomes following the folding and departure of CFTR from the ER.

Proteins located in the endoplasmic reticulum form the first set of protein partners that are primarily involved in synthesizing and folding CFTR [255,256]. Some of these partners play a role in ER quality control of CFTR, detecting misfolded channels and directing them for proteasomal degradation. ER quality control includes various checkpoints that involve chaperones, such as calreticulin, calnexin, Hsp70, and their co-chaperones [257]. The ATP-binding molecular chaperones Hsc70/Hsp70 and Hsp90 are crucial players in controlling the folding of CFTR [258,259,260]. These chaperones are aided by co-chaperones that assist in regulating the fate of CFTR by evaluating its folding and distinguishing between folded and misfolded proteins. For instance, HOP, which is a protein responsible for organizing Hsc70/Hsp90, promotes CFTR degradation by facilitating interactions with CHIP, an E3 ubiquitin ligase that catalyzes the process of CFTR ubiquitination. HspBP1 is among the co-factors that promote folding and functions as a nucleotide exchange factor for Hsc70/Hsp70. By counteracting its interaction with CHIP, HspBP1 prevents the degradation of CFTR [261]. Hsc70/Hsp70 co-chaperones, including Hsp40 proteins such as DNAJB9, DNAJB12, Hdj1, Hdj2, cysteine string protein, and several others, can either aid in folding or degradation [260,262,263,264,265,266]. This demonstrates the complex networks that regulate the quality control of CFTR in the ER. 

The Golgi apparatus plays a critical role in the glycosylation process of the CFTR protein, which is essential for its successful transportation to the plasma membrane. However, at the plasma membrane, not all CFTR proteins reach full maturation, and some are subjected to endocytosis and lysosomal degradation [267]. The C-terminus of CFTR contains a tyrosine-based motif, allowing for endocytosis, which is mediated by adaptor proteins like AP2 and Dab2 [268,269]. Endocytosed CFTR can either be recycled back to the cell surface or selected for lysosomal degradation [269,270]. The recycling pathway and membrane stability of CFTR are regulated by protein interactions with PDZ domain-containing proteins, the Rab family of GTPases, and myosin protein [256]. CAL, a PDZ scaffolding protein in the Golgi, binds to CFTR and transports it to lysosomes for degradation mediated by Syntaxin-6 [271]. Other syntaxins also regulate CFTR function and trafficking. Syntaxin 1A, for example, interacts with CFTR and inhibits its channel function, which can be competitively modulated by its interaction with Munc18 [103]. Disruption of syntaxin 1A interaction with F508del CFTR augmented mutant CFTR function [272]. Additionally, CFTR plays a vital role in lysosomal clearance, as Syntaxin 17, an autophagic SNARE protein, directly interacts with CFTR under the environmental stressors that induce autophagy [273]. Syntaxin 17 incorporates CFTR into mature autophagosomes to promote effective lysosomal clearance. Impaired CFTR function results in the absence of CFTR-Stx17 interaction, affecting bacterial clearance. Therefore, the interaction between CFTR and syntaxin 17 is expected to be a crucial regulator of autophagy, especially during infectious diseases, lysosomal clearance disorders, and in CF airways [273]. Perturbed CFTR-Stx17 interactions in CF may underlie the poor autophagy that has been consistently reported in CF [274,275]. Once at the cell surface, CFTR channel activity is mainly regulated by phosphorylation of its regulatory domain. Several kinases participate in regulating this process, primarily PKA [276,277], followed by PKC [278,279], and tyrosine kinases [280]. Recently, Mihalyi et al. showed that CFTR association with PKA initiated conformational changes leading to channel activation [102]. The phosphorylation of specific residues is necessary for maintaining its effects over an extended period.

Several PDZ proteins are reported to interact with the finally placed CFTR at the plasma membrane. PDZ domains are 80–90 amino acid residue-long protein–protein interaction domains that typically help anchor the correct targets to the cytoskeleton [281]. PDZ-based protein–protein interactions regulate the biosynthesis, processing, and trafficking of CFTR. NHERF1 (Na/H exchanger regulatory factor (1), NHERF2, PDZK1 (PDZ domain-containing protein in kidney 1), PDZK2, and Shank2 (SH3 and Ankyrin repeat-containing protein (2) are some of the PDZ scaffolding proteins that bind to the carboxyl-terminal tail of CFTR [14,81]. Several macromolecular complexes comprising CFTR have been identified on the apical plasma membrane of epithelial cells. These include the β_2_AR-NHERF1-CFTR, MRP4-PDZK1-CFTR, LPA_2_R-NHERF2-CFTR, and iNOS-NHERF2-CFTR complexes that are expected to form in many CF-relevant epithelial cells [282,283,284,285,286]. Additionally, NHERF1 is known to associate CFTR with a cAMP-sensitive cytoskeletal-anchoring complex with ezrin and PKA [287]. In general, these PDZ interactions become orchestrated to regulate CFTR in a highly compartmentalized manner in response to various tissue-specific stimuli and in the context of normal and pathological conditions. 

CFTR present on the cell membrane also exists within the complexes containing the enzymes adenylate and guanylate cyclases that directly provide the activating stimuli of cAMP and cGMP, respectively [288]. These molecular couplings are known to be associated with the pathological occurrence of secretory diarrhea but may bear several key normal physiological roles that are still evolving. Luminal exposure to bacterial enterotoxins in the gut stimulates excessive production of cAMP and cGMP, which act as secretory impetus for CFTR [289]. Increased Cl^−^ secretions through CFTR across the epithelial membranes lead to water transit coupled with an increase in the electrical and osmotic drive. The overstimulated fluid secretion due to hyperactive CFTR chloride channels then manifests as diarrhea [290]. Infectious diarrhea is a significant public health issue worldwide that is ranked as the second highest cause of death among children under five years of age [291,292]. Heat-labile cholera toxin secreted by *Vibrio cholerae* causes constitutive activation of the Gαs pathway that results in excessive synthesis of intracellular cAMP through AC6 [293,294]. The heat-stable *E. coli* and *Y. enterocolitica* toxins stimulate excessive synthesis of intracellular cGMP. Both cAMP and cGMP activate CFTR chloride channels by triggering phosphorylation of the R domain in CFTR [295,296]. Phosphodiesterase (PDE), which catalyzes the hydrolysis of cAMP and cGMP, becomes an important modulator of local pools of cAMP and cGMP for CFTR activation and through further putative interactions [297,298]. In this context, we would like to highlight two key interacting proteins of CFTR, Adenylate cyclase 6 (AC6) and Guanylate cyclase 2C (GCC), that act as a direct activating source for CFTR by supplying cAMP and cGMP, respectively, and at the same time, keep the signaling highly compartmentalized and specific. 

### 1.12. AC6

**Figure 4:**
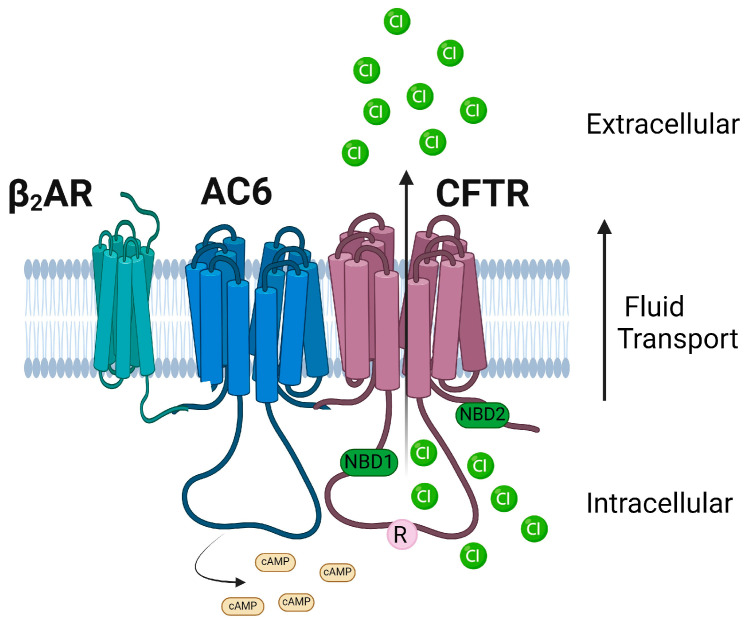
Adenylate cyclase 6 contains macromolecular complexes of CFTR.

We and others recently discovered that AC6 is the most dominant form of adenylate cyclase among the ten known adenylate cyclases in the airway and intestinal epithelium [299]. Furthermore, AC6 binds CFTR and is required for cAMP-dependent activation of CFTR. Two independent studies determined that AC6 function and CFTR-AC6 macromolecular complex formation are central to the development of diarrhea upon cholera toxin challenge [299,300]. Consequently, fluid secretion in the ileum in response to cholera toxin was largely absent in mice upon epithelium-specific removal of AC6 expression, while cGMP elicited secretion though heat-stable toxin analog Linaclotide was preserved. Small molecules that target AC6 or AC6 containing macromolecular complexes are potentially an attractive therapy to rapidly treat diarrhea or could benefit conditions of constipation. According to the hypothesis of PKA binding to CFTR as essential for CFTR channel opening [102] and evidence of AC6 as the key cAMP source for CFTR activation, coupling of AC6 to PKA could provide important regulatory and protein binding cues for CFTR function in the epithelium [299]. The process of compartmentalization of cAMP within small cellular microdomains could make CFTR activation and inhibition highly specific and sensitive to low-amplitude signaling events (Figure 4) [298,301].

A unique role of AC6 emerged in the mucociliary transport process through the regulation of ciliary function [302]. AC6 was demonstrated to regulate ciliary kinesin Kif19a via autophagy and, through this process, control the height of the cilia. Loss of AC6 resulted in a higher rate of degradation of Kif19a and longer cilia via downregulation of mTOR and upregulation of AMPK signaling pathways. The activation of the AMPK pathway causes the mobilization of Kif19a into autophagosomes, leading to its degradation. The study, for the first time, established the inherent plasticity in motile cilia that could be an important determinant for the amplitude of ciliary signaling and regulate the process of mucociliary transport in CF airways. Based on our unpublished data, AC6 also regulates ciliary beat frequency with loss of AC6, resulting in significant depression of the ciliary beat rate. Through these roles, AC6 is being established as an important player in regulating mucociliary clearance in the airways. Based on the data from the open-source LungMAP single-cell reference, AC6 expression is highly enriched in secretory cells (mucous cells) and basal cells, with a significant expression in ciliated cells and ionocytes [212]. Considering the dominant contribution of secretory cells to CFTR function in the airway epithelium and the fact that CFTR and AC6 can interact with each other [299], this CFTR-AC6 complex formation (in secretory cells) could be central to the activation of CFTR and fluid balance in the airways. Ionocytes are distinctly enriched in AC5, consistent with the increased expression of AC5 in AAV-*FOXI1* transduced ionocyte-enriched airway cultures [206].

### 1.13. GCC

GCC is a predominantly expressed receptor in the intestinal epithelial cells for hormones such as uroguanylin and guanylin, along with a heat-stable enterotoxin from *E*. *coli* (STa) [303]. GCC activation enhances the production of cGMP, which further increases chloride transport to drive electrolyte and fluid transport into the intestinal lumen [304,305]. Mutation in GCC is linked to MI, or obstructed bowel, and diarrhea [306,307]. Additionally, the GCC agonist, Linaclotide (LC), is clinically proven to improve constipation in Irritable Bowel Syndrome (IBS) patients [308]. How exposure to GCC activators impacts mutant CFTR function was investigated by our laboratory [309]. STcore (STC) is a GCC-agonist peptide containing 14 amino acids like LC that is used clinically for IBS. We demonstrated that STC increased the maturation of F508del-CFTR and promoted functional rescue of different mutant forms of CFTR. The benefit of GCC agonists in the functional rescue of CFTR was demonstrated in CF patient-derived intestinal organoids with various combinations of CFTR mutations [309]. The discovery of this unconventional role of GCC in facilitating improved folding of CFTR, irrespective of the mutation type, might underlie the general nature of the mild pathology of the GI system in CF.

## 2. Summary

**Figure 5:**
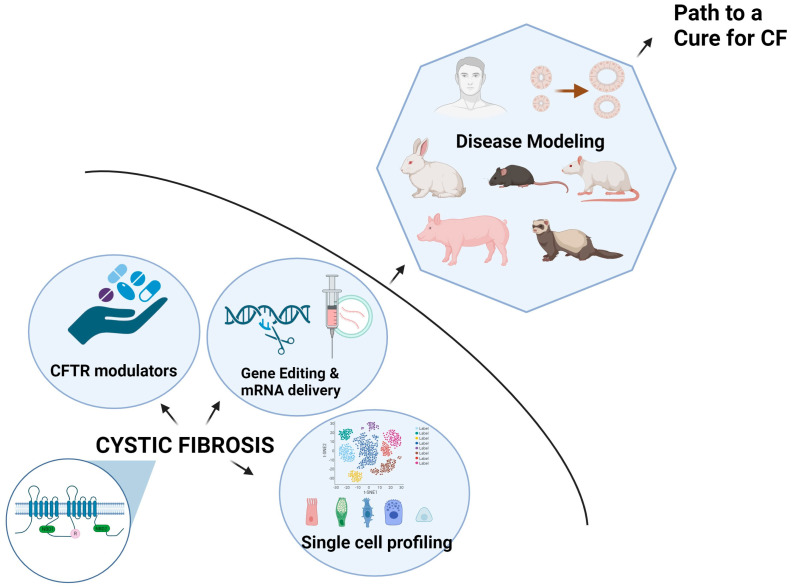
Current active strategies for cystic fibrosis therapy.

CF is caused by a single gene defect in CFTR. By means of the tremendous efforts that went into understanding the molecular, structural, and functional characteristics of CFTR, leading to the breakthrough treatments that we have today, CF is an exemplary model of how to approach and study monogenic diseases. CFTR is part of a wide network of signaling complexes that play a critical role in health and disease. Such complexes become critical in the context of how highly effective CFTR modulators provide benefits for CF, non-CF, CFTR-related disorders, and CF-like disorders. However, there is still a big knowledge gap, especially in terms of the changes in the transcriptome, proteome, and secretome that happen at the level of complex interactions between cell-to-cell and cell-to-environment in CF. Currently, there are ongoing, highly resolved studies of the molecular compositions of different cells and tissue microenvironments that will help us better understand CF. The hope is that these studies will direct us toward devising therapies for other aspects of CF disease that are not currently addressed by existing therapies and the CF in complete non-responders and ineligible patients while making progress toward the path for precision medicine in CF and cure for all (Figure 5).
